# Crystal structure of *catena*-poly[[di­aqua(4,5-di­aza­fluoren-9-one-κ^2^ *N*,*N*′)cadmium]-μ-2-hydroxy-5-sulfonato­benzoato-κ^3^ *O* ^1^,*O* ^1′^:*O* ^5^]

**DOI:** 10.1107/S1600536814023472

**Published:** 2014-10-31

**Authors:** Chun-Xiang Wang, Zhi-Feng Li

**Affiliations:** a School of Materials Science and Engineering, Jiangxi University of Science and Technology, Ganzhou 341000, People’s Republic of China

**Keywords:** crystal structure, one-dimensional coordination polymer, 4,5-di­aza­fluoren-9-one, 2-hy­droxy-5-sulfonato­benzoate, cadmium complex, hydrogen bonding

## Abstract

In the polymeric title compound, [Cd(C_7_H_4_O_6_S)(C_11_H_6_N_2_O)(H_2_O)_2_]_*n*_, the Cd^2+^ atom is seven-coordinated by two water O atoms, by three O atoms from two 2-hy­droxy-5-sulfonato­benzoate (Hssal^2−^) ligands and by two N atoms from a 4,5-di­aza­fluoren-9-one (Dafo) ligand in a distorted penta­gonal–bipyramidal geometry. The Cd^2+^ atoms are monodentately coordinated by the sulfonate group of one Hssal^2−^ ligand and bidentately coordinated by the carboxyl­ate group of another Hssal^2−^ ligand, generating zigzag chains running parallel to [010]. The chains are linked by O—H⋯O hydrogen bonds into a three-dimensional architecture.

## Related literature   

For information on compounds with metal–organic framework structures, see: Song *et al.* (2007 ▶); Yan *et al.* (2009 ▶). For related Cd^2+^ compounds, see: Sun *et al.* (2010 ▶). 
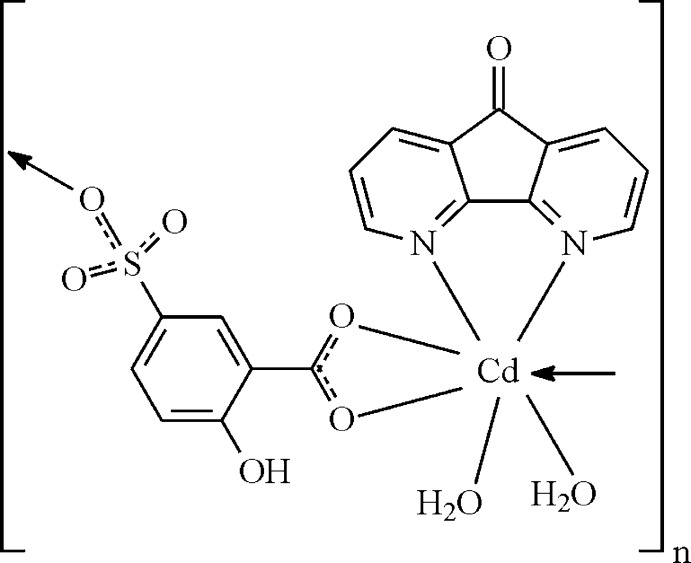


## Experimental   

### Crystal data  

[Cd(C_7_H_4_O_6_S)(C_11_H_6_N_2_O)(H_2_O)_2_]
*M* *_r_* = 546.77
Monoclinic, 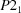

*a* = 7.6233 (6) Å
*b* = 12.8461 (10) Å
*c* = 9.4625 (7) Å
β = 98.155 (1)°
*V* = 917.29 (12) Å^3^
*Z* = 2
Mo *K*α radiation
μ = 1.37 mm^−1^
*T* = 296 K
0.35 × 0.32 × 0.20 mm

### Data collection  

Bruker SMART APEXII CCD area-detector diffractometer
Absorption correction: multi-scan (*SADABS*; Sheldrick, 2003 ▶) *T*_min_ = 0.677, *T*_max_ = 0.823
5158 measured reflections
3545 independent reflections
3469 reflections with *I* > 2σ(*I*)
*R*_int_ = 0.010

### Refinement  

*R*[*F* ^2^ > 2σ(*F* ^2^)] = 0.015
*wR*(*F* ^2^) = 0.037
*S* = 1.02
3545 reflections
281 parameters
1 restraint
H-atom parameters constrained
Δρ_max_ = 0.32 e Å^−3^
Δρ_min_ = −0.23 e Å^−3^
Absolute structure: Flack (1983 ▶), 1559 Friedel pairs
Absolute structure parameter: 0.040 (13)

### 

Data collection: *APEX2* (Bruker, 2004 ▶); cell refinement: *SAINT* (Bruker, 2004 ▶); data reduction: *SAINT*; program(s) used to solve structure: *SHELXS97* (Sheldrick, 2008 ▶); program(s) used to refine structure: *SHELXL97* (Sheldrick, 2008 ▶); molecular graphics: *SHELXTL* (Sheldrick, 2008 ▶); software used to prepare material for publication: *SHELXTL*.

## Supplementary Material

Crystal structure: contains datablock(s) I, New_Global_Publ_Block. DOI: 10.1107/S1600536814023472/im2455sup1.cif

**Fig. 1.**
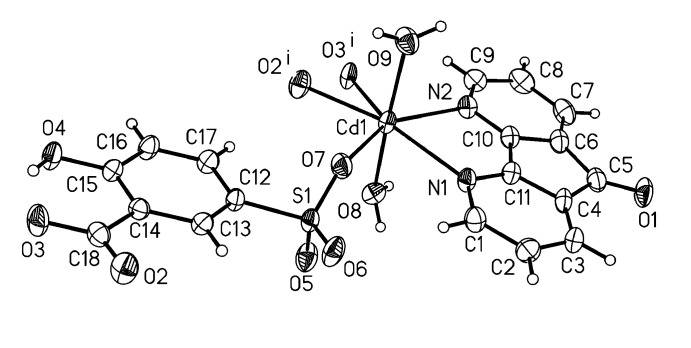
The asymmetric unit of the title compounds showing displacement ellipsoids at the 30% probability level and H atoms as small spheres of arbitrary radii. Symmetry code: (i) 1 - x, 0.5 + y, 2 - z.

Structure factors: contains datablock(s) I. DOI: 10.1107/S1600536814023472/im2455Isup2.hkl

x y z . DOI: 10.1107/S1600536814023472/im2455fig1.tif
The asymmetric unit of the title compounds showing displacement ellipsoids at the 30% probability level and H atoms as small spheres of arbitrary radii. Symmetry code: (i) 1 − *x*, 0.5 + *y*, 2 − *z*.

**Fig. 2.**
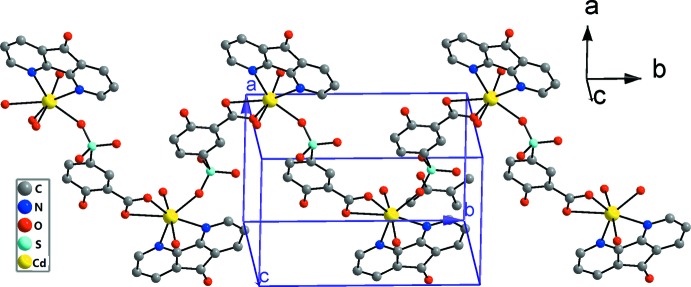
The one-dimensional zigzag-like chain structure of the title compound. H atoms are omitted for clarity.

. DOI: 10.1107/S1600536814023472/im2455fig2.tif
The one-dimensional zigzag-like chain structure of the title compound. H atoms are omitted for clarity.

CCDC reference: 1030993

Additional supporting information:  crystallographic information; 3D view; checkCIF report

## Figures and Tables

**Table 1:** Hydrogen-bond geometry (, )

*D*H*A*	*D*H	H*A*	*D* *A*	*D*H*A*
O8H8*A*O6^i^	0.85	1.96	2.801(3)	171
O8H8*B*O4^ii^	0.85	1.93	2.758(3)	164
O9H9*A*O5^iii^	0.85	1.98	2.771(3)	154
O9H9*B*O1^iv^	0.85	2.00	2.820(3)	161

Symmetry codes: (i) 
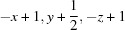
; (ii) 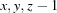; (iii) 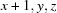; (iv) 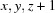.

## supplementary crystallographic information

### S1. Comment

In order to understand the coordination chemistry of H_3_ssal and to prepare new supramolecular materials with intriguing structures and potential physical properties, 5-sulfosalicylic acid and chelating bipyridyl-like ligands were widely used to construct coordination complexes (Yan *et al.*, 2009). Nevertheless, the Dafo ligand was seldom referred as one of these bipyridyl-like ligands. We chose 2-hydroxy-5-sulfobenzoic acid as an organic carboxylate anion and 4,5-diazafluoren-9-one as a neutral ligand with a N_2_-donor set to generate the coordination compound, [Cd(C_11_H_6_N_2_O)(C_7_H_4_O_6_S)(H_2_O)_2_]_n_, which is reported here, under hydrothermal conditions.

As shown in Fig. 1, the asymmetric unit of the title compound consists of one Cd^2+^ cation, one Hssal^2-^ anion, one Dafo ligand and two aqua ligands. Each Cd(II) center adopts a distorted pentagonal bipyramidal geometry and is seven-coordinated by one sulfonato oxygen atom and two carboxylate oxygen atoms from two different Hssal^2-^ ligands, two N atoms from one Dafo molecule and two water O atoms. The oxygen atoms from the Hssal^2-^ ligands and the nitrogen atoms form the basal plane while the axial positions are occupied by two water oxygen atoms. The Cd—O distances are in the range of 2.285 (2) - 2.629 (2) Å, with an average bond length of 2.3736 (2) Å, which are all within the normal range generally found in the literature (Sun *et al.*, 2010). Hssal^2-^ functions as a tridentate ligand, in which two carboxylate oxygen atoms chelate one Cd atom and one sulfonato oxygen atom binds to another Cd atom. Thus the Cd(II) centers are bridged by the Hssal^2-^ ligands to generate one-dimensional zigzag chains along [010]. Moreover, an extensive hydrogen bond network is observed in the crystal structure of the title compound in which the aqua ligands (O8 and O9) act as the hydrogen donors towards sulfonato oxygen atoms O5 and O6, the hydroxyl oxygen atom O4 and the ketone oxygen atom O1, respectively.

### S2. Experimental

A mixture of Cd(NO_3_)_2_ × 4 H_2_O (1.00 mmol, 0.3085 g), 5-sulfosalicylic acid (H_3_ssal) dihydrate (1 mmol, 0.2542 g), 4,5-diazafluoren-9-one (Dafo) (1 mmol, 0.1822 g), NaOH (1.00 mmol, 0.04 g) and H_2_O (10.0 ml) was heated in a 23 ml Teflon-lined stainless steel reactor at 443 K for 72 h. The yellow plate-like crystals were filtered and washed with water and acetone. Yield: 8% based on Cd.

### S3. Refinement

H atoms attached to C atoms were included at calculated positions and treated as riding atoms [C—H = 0.93 Å and *U*_iso_(H) = 1.2 *U*_eq_(C)]. Water H atoms were found in a difference map, relocated in idealized positions (O—H = 0.85 Å) and refined as riding atoms with *U*_iso_(H) = 1.5 *U*_eq_(O). The highest density peak is located 0.85 Å from atom Cd and the deepest hole is located 0.69 Å from atom S.

### Crystal data

**Table:**

[Cd(C_7_H_4_O_6_S)(C_11_H_6_N_2_O)(H_2_O)_2_]	*F*(000) = 544
*M**_r_* = 546.77	*D*_x_ = 1.980 Mg m^−^^3^
Monoclinic, *P*2_1_	Mo *K*α radiation, λ = 0.71073 Å
Hall symbol: P 2yb	Cell parameters from 387 reflections
*a* = 7.6233 (6) Å	θ = 2.1–27.5°
*b* = 12.8461 (10) Å	µ = 1.37 mm^−^^1^
*c* = 9.4625 (7) Å	*T* = 296 K
β = 98.155 (1)°	Plate, yellow
*V* = 917.29 (12) Å^3^	0.35 × 0.32 × 0.20 mm
*Z* = 2	

### Data collection

**Table:**

Bruker SMART APEXII CCD area-detector diffractometer	3545 independent reflections
Radiation source: fine-focus sealed tube	3469 reflections with *I* > 2σ(*I*)
Graphite monochromator	*R*_int_ = 0.010
φ and ω scans	θ_max_ = 26.7°, θ_min_ = 2.2°
Absorption correction: multi-scan (*SADABS*; Sheldrick, 2003)	*h* = −9→8
*T*_min_ = 0.677, *T*_max_ = 0.823	*k* = −16→16
5158 measured reflections	*l* = −9→11

### Refinement

**Table:**

Refinement on *F*^2^	Hydrogen site location: inferred from neighbouring sites
Least-squares matrix: full	H-atom parameters constrained
*R*[*F*^2^ > 2σ(*F*^2^)] = 0.015	*w* = 1/[σ^2^(*F*_o_^2^) + (0.0198*P*)^2^] where *P* = (*F*_o_^2^ + 2*F*_c_^2^)/3
*wR*(*F*^2^) = 0.037	(Δ/σ)_max_ = 0.010
*S* = 1.02	Δρ_max_ = 0.32 e Å^−^^3^
3545 reflections	Δρ_min_ = −0.23 e Å^−^^3^
281 parameters	Extinction correction: *SHELXL97* (Sheldrick, 2008), Fc^*^=kFc[1+0.001xFc^2^λ^3^/sin(2θ)]^-1/4^
1 restraint	Extinction coefficient: 0.0055 (5)
Primary atom site location: structure-invariant direct methods	Absolute structure: Flack (1983), 1559 Friedel pairs
Secondary atom site location: difference Fourier map	Absolute structure parameter: 0.040 (13)

### Special details

**Table:**

Geometry. All e.s.d.'s (except the e.s.d. in the dihedral angle between two l.s. planes) are estimated using the full covariance matrix. The cell e.s.d.'s are taken into account individually in the estimation of e.s.d.'s in distances, angles and torsion angles; correlations between e.s.d.'s in cell parameters are only used when they are defined by crystal symmetry. An approximate (isotropic) treatment of cell e.s.d.'s is used for estimating e.s.d.'s involving l.s. planes.
Refinement. Refinement of *F*^2^ against ALL reflections. The weighted *R*-factor *wR* and goodness of fit *S* are based on *F*^2^, conventional *R*-factors *R* are based on *F*, with *F* set to zero for negative *F*^2^. The threshold expression of *F*^2^ > σ(*F*^2^) is used only for calculating *R*-factors(gt) *etc*. and is not relevant to the choice of reflections for refinement. *R*-factors based on *F*^2^ are statistically about twice as large as those based on *F*, and *R*- factors based on ALL data will be even larger.

### Fractional atomic coordinates and isotropic or equivalent isotropic displacement parameters (Å^2^)

**Table:**

	*x*	*y*	*z*	*U*_iso_*/*U*_eq_	
C1	0.7119 (3)	0.11149 (18)	0.4387 (2)	0.0325 (5)	
H1	0.6659	0.0842	0.5167	0.039*	
C2	0.7228 (3)	0.04706 (19)	0.3233 (3)	0.0369 (5)	
H2	0.6851	−0.0217	0.3254	0.044*	
C3	0.7902 (3)	0.08485 (19)	0.2036 (3)	0.0377 (5)	
H3	0.7993	0.0427	0.1251	0.045*	
C4	0.8423 (3)	0.18736 (18)	0.2071 (2)	0.0305 (5)	
C5	0.9171 (3)	0.2583 (2)	0.1039 (3)	0.0349 (5)	
C6	0.9390 (2)	0.3622 (3)	0.17581 (19)	0.0327 (4)	
C7	1.0001 (3)	0.4579 (2)	0.1391 (3)	0.0412 (6)	
H7	1.0365	0.4693	0.0506	0.049*	
C8	1.0049 (3)	0.5374 (2)	0.2407 (3)	0.0434 (6)	
H8	1.0489	0.6026	0.2215	0.052*	
C9	0.9445 (3)	0.51923 (17)	0.3698 (3)	0.0355 (5)	
H9	0.9428	0.5746	0.4329	0.043*	
C10	0.8864 (2)	0.3516 (2)	0.3100 (2)	0.0280 (4)	
C11	0.8275 (3)	0.24562 (16)	0.3282 (2)	0.0282 (4)	
C12	0.4313 (3)	0.19306 (15)	0.9233 (2)	0.0248 (4)	
C13	0.3771 (3)	0.11221 (16)	1.0031 (2)	0.0261 (4)	
H13	0.3678	0.0454	0.9650	0.031*	
C14	0.3361 (3)	0.12964 (15)	1.1403 (2)	0.0241 (4)	
C15	0.3489 (3)	0.23148 (16)	1.1954 (2)	0.0262 (4)	
C16	0.4067 (3)	0.31269 (17)	1.1150 (2)	0.0308 (5)	
H16	0.4169	0.3797	1.1525	0.037*	
C17	0.4488 (3)	0.29364 (16)	0.9797 (2)	0.0291 (5)	
H17	0.4885	0.3475	0.9267	0.035*	
C18	0.2836 (3)	0.04158 (16)	1.2294 (2)	0.0277 (4)	
Cd1	0.760273 (16)	0.360333 (13)	0.613628 (12)	0.02682 (5)	
N1	0.7645 (2)	0.21201 (14)	0.44437 (19)	0.0281 (4)	
N2	0.8881 (2)	0.42577 (14)	0.4086 (2)	0.0301 (4)	
O1	0.9547 (2)	0.23396 (15)	−0.01156 (18)	0.0460 (4)	
O2	0.2854 (2)	−0.04920 (12)	1.18426 (18)	0.0392 (4)	
O3	0.2427 (2)	0.06334 (13)	1.35128 (17)	0.0377 (4)	
O4	0.3065 (2)	0.25331 (12)	1.32612 (16)	0.0344 (4)	
H4	0.2813	0.1972	1.3664	0.052*	
O5	0.3267 (2)	0.22249 (15)	0.65477 (18)	0.0418 (4)	
O6	0.4806 (2)	0.06102 (12)	0.72661 (17)	0.0394 (4)	
O7	0.6409 (2)	0.22356 (13)	0.73467 (17)	0.0359 (4)	
O8	0.4984 (2)	0.40770 (12)	0.48081 (17)	0.0338 (3)	
O9	1.0370 (2)	0.32540 (13)	0.73495 (18)	0.0411 (4)	
S1	0.47242 (7)	0.17302 (4)	0.74579 (5)	0.02593 (11)	
H8B	0.4229	0.3687	0.4315	0.039*	
H8A	0.5159	0.4564	0.4233	0.039*	
H9B	1.0268	0.3091	0.8205	0.039*	
H9A	1.1283	0.3117	0.6959	0.039*	

### Atomic displacement parameters (Å^2^)

**Table:**

	*U*^11^	*U*^22^	*U*^33^	*U*^12^	*U*^13^	*U*^23^
C1	0.0372 (12)	0.0294 (11)	0.0329 (12)	0.0010 (9)	0.0118 (9)	0.0016 (9)
C2	0.0391 (13)	0.0270 (11)	0.0451 (15)	−0.0021 (9)	0.0082 (10)	−0.0056 (11)
C3	0.0377 (12)	0.0425 (13)	0.0347 (13)	0.0024 (10)	0.0114 (10)	−0.0125 (10)
C4	0.0337 (11)	0.0356 (12)	0.0240 (11)	0.0049 (9)	0.0107 (9)	−0.0025 (9)
C5	0.0299 (13)	0.0481 (14)	0.0279 (13)	0.0057 (10)	0.0082 (10)	0.0006 (10)
C6	0.0330 (9)	0.0392 (10)	0.0272 (9)	0.0066 (15)	0.0087 (7)	0.0022 (14)
C7	0.0398 (14)	0.0481 (15)	0.0389 (15)	0.0024 (12)	0.0163 (11)	0.0146 (11)
C8	0.0424 (13)	0.0349 (13)	0.0545 (16)	−0.0024 (10)	0.0125 (11)	0.0125 (11)
C9	0.0366 (12)	0.0270 (11)	0.0434 (14)	0.0001 (9)	0.0071 (10)	0.0021 (10)
C10	0.0302 (9)	0.0285 (11)	0.0262 (9)	0.0045 (11)	0.0072 (7)	0.0026 (11)
C11	0.0319 (11)	0.0293 (11)	0.0241 (11)	0.0025 (8)	0.0067 (9)	−0.0001 (9)
C12	0.0305 (10)	0.0242 (10)	0.0207 (10)	−0.0005 (8)	0.0074 (8)	−0.0005 (8)
C13	0.0307 (10)	0.0236 (9)	0.0240 (10)	−0.0010 (8)	0.0036 (8)	0.0008 (8)
C14	0.0245 (10)	0.0249 (10)	0.0228 (10)	−0.0005 (7)	0.0031 (8)	0.0026 (8)
C15	0.0296 (10)	0.0283 (10)	0.0207 (10)	0.0004 (8)	0.0029 (8)	−0.0002 (8)
C16	0.0447 (12)	0.0220 (9)	0.0269 (11)	−0.0046 (9)	0.0090 (9)	−0.0044 (9)
C17	0.0397 (12)	0.0227 (10)	0.0256 (11)	−0.0037 (8)	0.0071 (9)	0.0032 (8)
C18	0.0285 (11)	0.0284 (11)	0.0266 (11)	0.0005 (9)	0.0053 (8)	0.0068 (9)
Cd1	0.03529 (8)	0.02431 (7)	0.02215 (7)	0.00261 (8)	0.00855 (5)	−0.00201 (7)
N1	0.0358 (10)	0.0268 (9)	0.0236 (9)	−0.0001 (7)	0.0108 (7)	0.0002 (7)
N2	0.0338 (10)	0.0278 (9)	0.0300 (10)	0.0024 (7)	0.0088 (8)	0.0016 (7)
O1	0.0552 (11)	0.0605 (11)	0.0258 (9)	0.0062 (9)	0.0179 (8)	−0.0043 (8)
O2	0.0571 (11)	0.0267 (8)	0.0356 (10)	−0.0041 (7)	0.0131 (8)	0.0045 (7)
O3	0.0517 (10)	0.0372 (9)	0.0275 (8)	−0.0024 (7)	0.0170 (7)	0.0068 (7)
O4	0.0516 (10)	0.0311 (8)	0.0230 (8)	−0.0039 (7)	0.0136 (7)	−0.0024 (6)
O5	0.0477 (10)	0.0529 (11)	0.0247 (9)	0.0116 (9)	0.0054 (7)	0.0019 (8)
O6	0.0644 (11)	0.0250 (8)	0.0316 (9)	−0.0041 (7)	0.0169 (8)	−0.0068 (6)
O7	0.0396 (9)	0.0381 (9)	0.0330 (9)	−0.0065 (7)	0.0155 (7)	−0.0005 (7)
O8	0.0406 (8)	0.0270 (7)	0.0325 (9)	−0.0018 (7)	0.0012 (7)	0.0018 (7)
O9	0.0377 (9)	0.0494 (11)	0.0376 (9)	0.0065 (7)	0.0103 (7)	0.0094 (7)
S1	0.0351 (3)	0.0234 (2)	0.0208 (2)	−0.0010 (2)	0.0093 (2)	−0.00040 (19)

### Geometric parameters (Å, º)

**Table:**

C1—N1	1.351 (3)	C14—C15	1.407 (3)
C1—C2	1.382 (3)	C14—C18	1.499 (3)
C1—H1	0.9300	C15—O4	1.351 (3)
C2—C3	1.395 (4)	C15—C16	1.399 (3)
C2—H2	0.9300	C16—C17	1.385 (3)
C3—C4	1.375 (3)	C16—H16	0.9300
C3—H3	0.9300	C17—H17	0.9300
C4—C11	1.386 (3)	C18—O2	1.243 (3)
C4—C5	1.506 (3)	C18—O3	1.268 (3)
C5—O1	1.209 (3)	Cd1—O8	2.2857 (16)
C5—C6	1.497 (4)	Cd1—O9	2.2984 (16)
C6—C7	1.376 (4)	Cd1—O2^i^	2.3061 (16)
C6—C10	1.391 (3)	Cd1—O7	2.3501 (16)
C7—C8	1.399 (4)	Cd1—N2	2.4404 (19)
C7—H7	0.9300	Cd1—N1	2.4922 (18)
C8—C9	1.385 (4)	Cd1—O3^i^	2.6294 (17)
C8—H8	0.9300	O2—Cd1^ii^	2.3061 (16)
C9—N2	1.344 (3)	O3—Cd1^ii^	2.6294 (17)
C9—H9	0.9300	O4—H4	0.8504
C10—N2	1.333 (3)	O5—S1	1.4518 (18)
C10—C11	1.452 (3)	O6—S1	1.4527 (16)
C11—N1	1.333 (3)	O7—S1	1.4561 (17)
C12—C13	1.381 (3)	O8—H8B	0.8500
C12—C17	1.397 (3)	O8—H8A	0.8519
C12—S1	1.771 (2)	O9—H9B	0.8507
C13—C14	1.395 (3)	O9—H9A	0.8510
C13—H13	0.9300		
			
N1—C1—C2	123.6 (2)	C16—C17—H17	120.2
N1—C1—H1	118.2	C12—C17—H17	120.2
C2—C1—H1	118.2	O2—C18—O3	122.4 (2)
C1—C2—C3	120.2 (2)	O2—C18—C14	119.85 (19)
C1—C2—H2	119.9	O3—C18—C14	117.73 (19)
C3—C2—H2	119.9	O8—Cd1—O9	174.22 (6)
C4—C3—C2	116.9 (2)	O8—Cd1—O2^i^	95.69 (6)
C4—C3—H3	121.6	O9—Cd1—O2^i^	85.41 (6)
C2—C3—H3	121.6	O8—Cd1—O7	95.66 (6)
C3—C4—C11	118.7 (2)	O9—Cd1—O7	90.12 (6)
C3—C4—C5	134.4 (2)	O2^i^—Cd1—O7	81.84 (6)
C11—C4—C5	107.0 (2)	O8—Cd1—N2	83.53 (6)
O1—C5—C6	128.0 (2)	O9—Cd1—N2	91.30 (6)
O1—C5—C4	126.1 (2)	O2^i^—Cd1—N2	127.58 (6)
C6—C5—C4	105.86 (19)	O7—Cd1—N2	150.56 (6)
C7—C6—C10	118.0 (3)	O8—Cd1—N1	86.57 (6)
C7—C6—C5	134.6 (2)	O9—Cd1—N1	94.42 (6)
C10—C6—C5	107.4 (2)	O2^i^—Cd1—N1	159.30 (6)
C6—C7—C8	117.1 (2)	O7—Cd1—N1	77.46 (6)
C6—C7—H7	121.5	N2—Cd1—N1	73.11 (6)
C8—C7—H7	121.5	O8—Cd1—O3^i^	77.46 (5)
C9—C8—C7	120.2 (2)	O9—Cd1—O3^i^	98.87 (6)
C9—C8—H8	119.9	O2^i^—Cd1—O3^i^	52.44 (5)
C7—C8—H8	119.9	O7—Cd1—O3^i^	132.00 (5)
N2—C9—C8	123.4 (2)	N2—Cd1—O3^i^	76.72 (5)
N2—C9—H9	118.3	N1—Cd1—O3^i^	147.20 (5)
C8—C9—H9	118.3	C11—N1—C1	114.62 (19)
N2—C10—C6	126.4 (3)	C11—N1—Cd1	108.76 (13)
N2—C10—C11	124.04 (19)	C1—N1—Cd1	136.50 (16)
C6—C10—C11	109.5 (2)	C10—N2—C9	114.8 (2)
N1—C11—C4	126.0 (2)	C10—N2—Cd1	110.21 (14)
N1—C11—C10	123.74 (19)	C9—N2—Cd1	134.77 (16)
C4—C11—C10	110.22 (19)	C18—O2—Cd1^ii^	100.22 (14)
C13—C12—C17	120.43 (19)	C18—O3—Cd1^ii^	84.48 (13)
C13—C12—S1	121.07 (15)	C15—O4—H4	109.5
C17—C12—S1	118.46 (16)	S1—O7—Cd1	140.81 (11)
C12—C13—C14	120.76 (19)	Cd1—O8—H8B	128.0
C12—C13—H13	119.6	Cd1—O8—H8A	110.0
C14—C13—H13	119.6	H8B—O8—H8A	103.7
C13—C14—C15	118.78 (18)	Cd1—O9—H9B	108.8
C13—C14—C18	121.01 (18)	Cd1—O9—H9A	124.9
C15—C14—C18	120.19 (18)	H9B—O9—H9A	123.6
O4—C15—C16	118.54 (19)	O5—S1—O6	113.71 (11)
O4—C15—C14	121.31 (19)	O5—S1—O7	111.74 (11)
C16—C15—C14	120.15 (19)	O6—S1—O7	112.26 (10)
C17—C16—C15	120.2 (2)	O5—S1—C12	106.01 (10)
C17—C16—H16	119.9	O6—S1—C12	106.20 (9)
C15—C16—H16	119.9	O7—S1—C12	106.27 (10)
C16—C17—C12	119.62 (19)		

Symmetry codes: (i) −*x*+1, *y*+1/2, −*z*+2; (ii) −*x*+1, *y*−1/2, −*z*+2.

### Hydrogen-bond geometry (Å, º)

**Table:**

*D*—H···*A*	*D*—H	H···*A*	*D*···*A*	*D*—H···*A*
O8—H8*A*···O6^iii^	0.85	1.96	2.801 (3)	171
O8—H8*B*···O4^iv^	0.85	1.93	2.758 (3)	164
O9—H9*A*···O5^v^	0.85	1.98	2.771 (3)	154
O9—H9*B*···O1^vi^	0.85	2.00	2.820 (3)	161

Symmetry codes: (iii) −*x*+1, *y*+1/2, −*z*+1; (iv) *x*, *y*, *z*−1; (v) *x*+1, *y*, *z*; (vi) *x*, *y*, *z*+1.
