# Supplementary figures and images for: Crystal structure of catena-poly[[di­aqua(4,5-di­aza­fluoren-9-one-κ2 N,N′)cadmium]-μ-2-hydroxy-5-sulfonato­benzoato-κ3 O 1,O 1′:O 5]
Source: Acta Crystallogr Sect E Struct Rep Online. 2014 Oct 31;70(Pt 11):m387–8. doi: 10.1107/S1600536814023472 (PMC4257252; doi:10.1107/S1600536814023472)

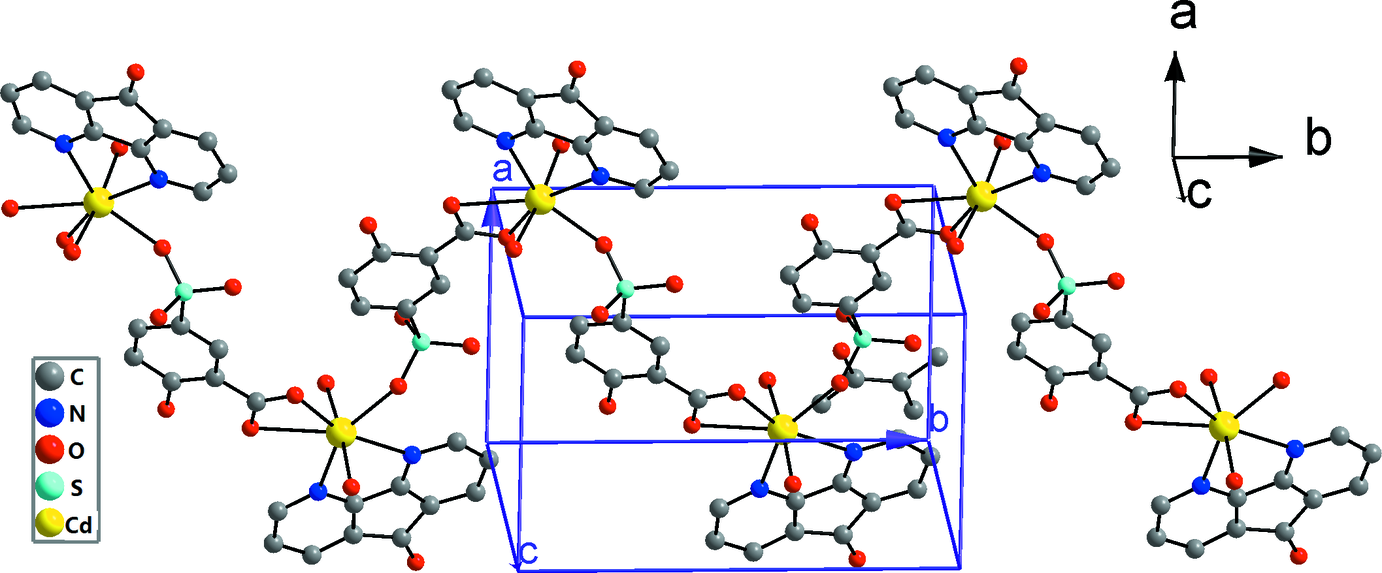

Supplement: Supplementary file 4 [file e-70-0m387-fig2.tif]
